# Minimal invasive interlaminar approach for untethering of fatty filum terminale in pediatric patients — how I do it

**DOI:** 10.1007/s00701-022-05204-y

**Published:** 2022-04-22

**Authors:** Ladina Greuter, Maria Licci, Axel Terrier, Raphael Guzman, Jehuda Soleman

**Affiliations:** 1 Department of Neurosurgery, University Hospital of Basel, Basel, Switzerland; 2 Department of Pediatric Neurosurgery, University Children’s Hospital of Basel, Basel, Switzerland; 3 Faculty of Medicine, University of Basel, Basel, Switzerland

**Keywords:** Fatty filum terminale, Filum terminale, Spinal dysraphism, Untethering, Tethered cord

## Abstract

**Background:**

Fatty filum terminale is a form of spinal dysraphism and a third of all patients develop symptoms such as sensory, motor, and urinary impairment. Early surgery at 6 months has the advantage that the bone density is still soft, and the patients are not ambulatory yet, promoting faster healing.

**Method:**

We present our minimal invasive surgical technique for FFT untethering.

**Conclusion:**

Due to the low complication rate and the potentially high benefit of surgery, prophylactic untethering is recommended.

**Supplementary Information:**

The online version contains supplementary material available at 10.1007/s00701-022-05204-y.

## Introduction

Fatty filum terminale (FFT) potentially results in a tethered cord. Approximately, a third of all patients with a FFT present with skin stigmata in the lumbosacral area [1], and can become symptomatic presenting with sensory or motor deficits, urinary incontinence or urgency, back and leg pain, and scoliosis. The classical treatment of FFT is a one to two level laminectomy and microsurgical untethering of the FFT, ideally at 6–9 months of age [6]. We present our technique of FFT untethering through a minimal invasive interlaminar approach and a small durotomy.

## Relevant surgical anatomy

The spinous process, which is often bifid in children < 6 years, lamina on each side of it, and facet joints are the bony landmarks. In small children, the disc space has approximately the same height as the vertebral body resulting in a larger interlaminar space, facilitating a minimal invasive approach. The filum terminale interna origins at the lower tip of the conus medullaris (CM) and continues to the end of the thecal sac [2]. Physiologically, the filum terminale has a high elasticity, which allows it to reduce elongation stress to the CM [2]. The increased fat content in a FFT reduces its elasticity, resulting in tension to the CM [9, 10]. The normal filum terminale diameter is 2 mm at the level L5/S1 and any larger diameter is considered pathological [5]. Typically, a FFT leads to a low-lying conus (under the level of L1) leading to a tethered cord syndrome. However, a normally positioned conus does not exclude the diagnosis of a FFT [10].

## Description of surgical technique

**Fig. 1:**
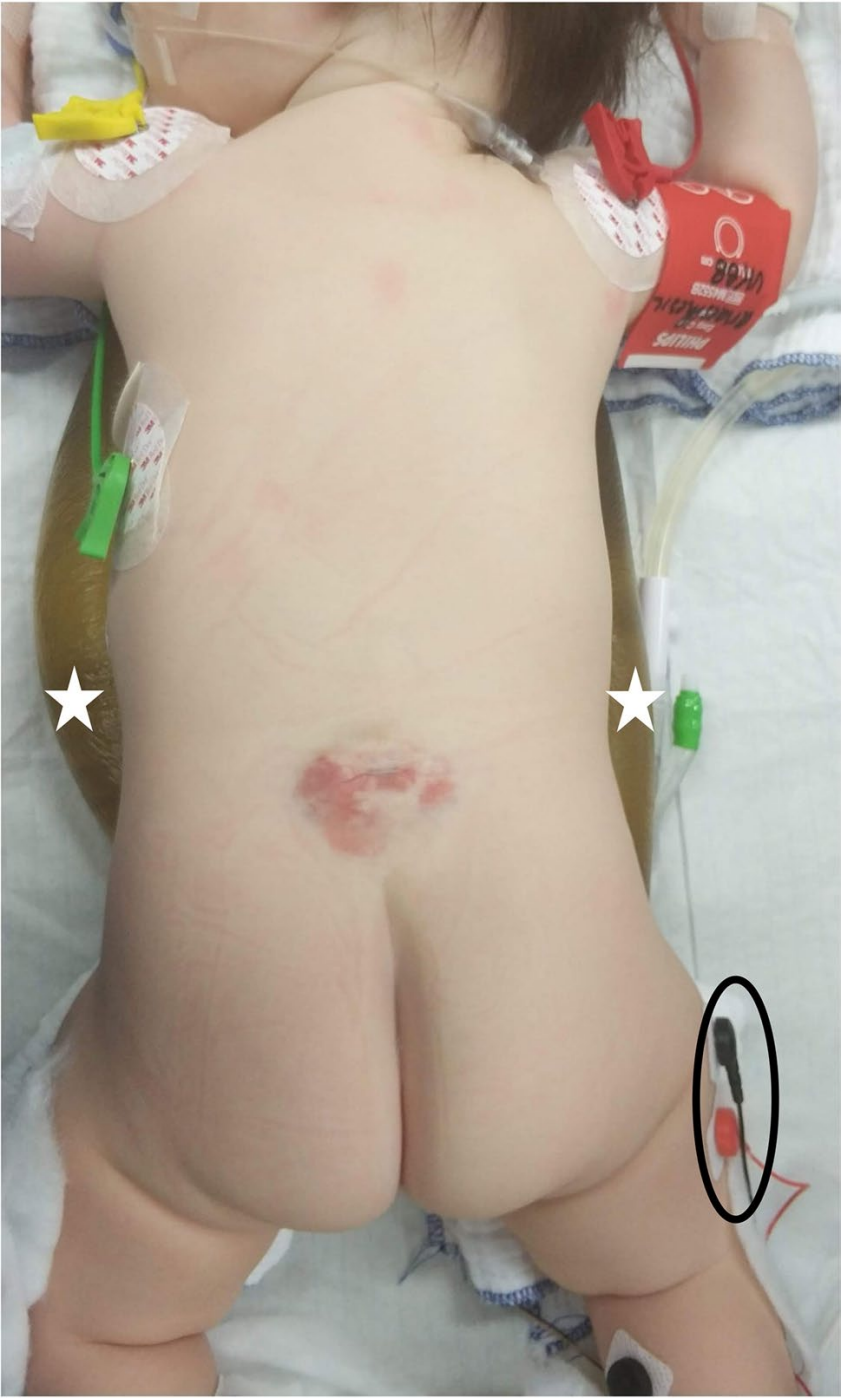
A 6-month-old female showing typical dermatological stigmata with a hemangioma and deviated gluteal fold. Preoperative prone positioning is shown with padding of all pressure points using jelly pads (white star) and IONM electrodes (black oval) in place (sphincter electrodes not placed yet)

**Fig. 2:**
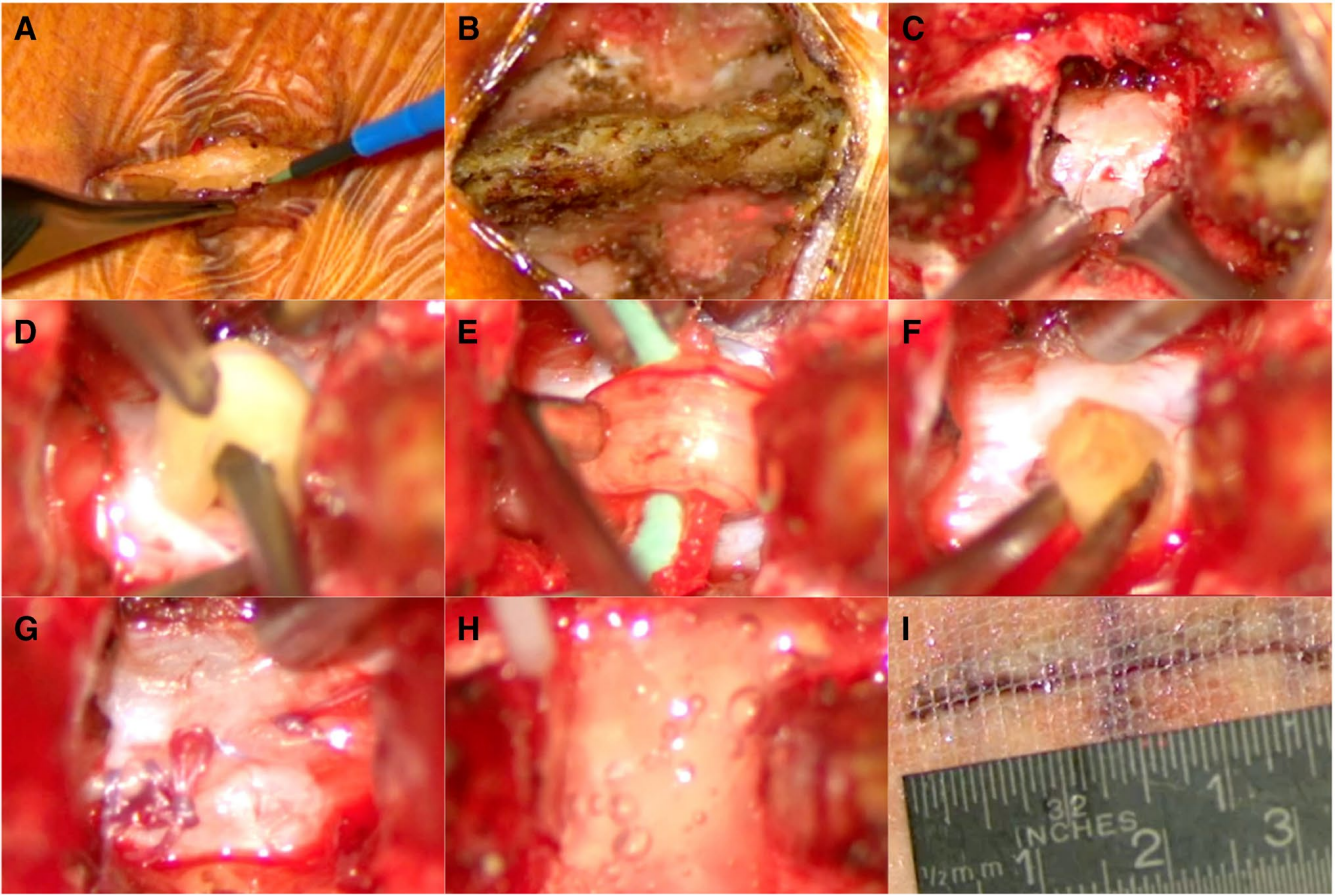
Surgical steps for fatty filum terminale untethering: **A** midline skin incision; **B** subperiosteal dissection of the muscle to expose the spinous process and lamina on both sides; **C** a small interlaminar approach is performed to visualize the dural sac; **D** dissection and careful mobilization of the fatty filum terminale; **E** electric stimulation of the fatty filum terminale before untethering; **F** dissected and cauterized cranial end of the fatty filum terminale; **G** watertight continuous dura closure with 4–0 PDS; **H** application of fibrin sealant (Tisseel, Baxter Inc.) to ensure dural closure; **I** skin closure of the small 3-cm-long incision with Dermabond Prineo® skin sealant system (Ethicon Inc., Raritan, NJ, USA)

Under general anesthesia, the patient is placed in prone position. Jelly rolls are positioned in a parallel fashion to the body from the mid clavicle to the iliac crest across the thorax to carefully pad all pressure points and relieve any abdominal pressure. Intraoperative neuromonitoring monitoring (IONM) with 14–16 muscles from L1 to S4 is recorded including anal sphincter electrodes for the bulbocavernosus reflex (Fig. 1). The level of incision is individually guided by the maximal point of fat within the filum. In infants, no routine fluoroscopy but anatomical landmarks are used to verify the level of incision. An approximately 3-cm-long midline incision is made (Fig. 2A). A subperiosteal dissection of the muscles on both sides exposing the laminae is conducted (Fig. 2B). The interspinous ligament is removed followed by a flavectomy. At times, very small portion of the laminae is resected (Fig. 2C). The interlaminar approach leads to a 1–1.5-cm dural exposure. Meticulous extradural hemostasis with coagulation, bone wax, and hemostatic agents (e.g., Floseal, Baxter International, Volketswil, Switzerland) is achieved. Using the surgical microscope, a midline durotomy of 0.8–1 cm is performed. The FFT is identified by its yellow appearance, and it is usually positioned in the midline (Fig. 2D). The FFT is dissected off the adjacent nerve roots and mobilized using a Rhoton microdissector. A small cotton patty is placed under the mobilized filum to keep it in place and create a barrier to the normal cauda equina nerve roots. Using a concentric bipolar stimulator probe with 0.2 milli Ampere (mA), the normal motor nerve roots are stimulated for quality control. Thereafter, the isolated FFT is stimulated with up to 10 mA while no motor response should be evoked (Fig. 2E). Bipolar diathermy is used to cauterize the FFT before transection. A small piece of FFT is sent for histopathological review for quality control. The cranial end is held in place with the bayonet forceps and its tip is thoroughly cauterized to avoid any bleeding (Fig. 2F). After its release, it often retracts rapidly cranially. The dura is closed in a watertight fashion using Ti-Cron™ (Medtronic, Minneapolis, MN, USA) 5–0 continuous suturing and fibrin sealant (TISSEEL, Baxter International, Volketswil, Switzerland) (Fig. 2G and H). Valsalva maneuver is performed to check for any CSF leak. The wound is closed in layers, using Vicryl 3–0 stitches for the fascia and Vicryl 4–0 stitches for the subcutaneous tissue. The skin is closed using an adhesive net and skin glue (Dermabond Prineo ®, Skin closure system, Ethicon Inc., Raritan, USA) (Fig. 2I, Video 1).

## Indications

Symptomatic patients diagnosed with FFT and regardless of the level of the CM should undergo surgery. No clear consensus exists for asymptomatic or oligosymptomatic patients with a FFT but normally placed CM or asymptomatic patients with an FFT and a low-lying CM [7, 8]. The advantage of prophylactic surgery (80% of all cases) is to avoid the development of neurologic deficits or scoliosis, which can occur in around 30% of the cases. The goal and reason of conducting the surgery must therefore be explained very thoroughly to the parents, for them to achieve an informed consent. Although controversial, we recommend surgery using IOM for quality control during surgery [4].

Surgery is ideally offered at the age of 6–9 months as it is early enough to avoid any neurologic damage, should they become symptomatic from the FFT, and has the advantage of a relatively soft bone, and less muscle tissue, facilitating surgery and recovery, allowing a minimal invasive approach, and reducing blood loss. Due to the low complication rate of around 2–4% and the potentially high benefit of surgery, we recommend prophylactic surgery to all of these children [3, 5].

## Limitations

Our minimal invasive approach has the limitation of a very small bone window and durotomy which at times might lead to difficulty in detecting the FFT.

## How to avoid complications

Even with IONM in place, careful dissection of the FFT from the cauda equina fibers needs to be ensured to avoid iatrogenic neurologic deficits. It is of paramount importance to ensure a watertight dura closure to prevent postoperative CSF leaks. This can either be achieved by continuous or interrupted suturing and possible addition of a sealant.

## Specific perioperative considerations

In our institute, we run an interdisciplinary clinic including pediatric neurosurgeons, neurologist, and urologist to assess these patients together. All patients should receive an MRI of the whole neuroaxis to exclude other concurrent pathologies (Chiari malformation, hydrocephalus, scoliosis). The level of the conus is best assessed in T2-weighted sequences, while the FFT is best visualized in non-fat-suppressed T1-weighted sequences.

In our practice, all patients receive a urinary catheter, which is removed 1–2 days after surgery, to prevent urinary retention, especially when patients are treated with morphine. After surgery, patients are kept in a horizontal position for 48 h to avoid a CSF fistula. As of the third postoperative day, babies should be showered, while the Dermabond Prineo® is removed 14 days after surgery in our outpatient clinic.

## Specific information for the patients about surgery and potential risks

The risk of possible neurological deficits despite the use of IONM or development CSF fistula is low (0–4%) [3, 5]. Re-tethering can occur in 2.7–8.6% of the cases and might indicate repeat surgery [5, 10]. The risk of wound infection is around 2–5% [3, 5]. In our experience, the overall risks and risk of postoperative CSF fistula are lower with a minimal invasive approach.

## Supplementary Information

Below is the link to the electronic supplementary material.

Supplementary file1 (MP4 83741 KB)
